# New Method of Inducing General Narcosis

**Published:** 1895-07

**Authors:** 


### NEW METHOD OF INDUCING GENERAL NARCOSIS.

Dr. Paul Rosenberg presented to the Berlin Medical Society a communication upon this subject, a report of which is found in the *Berliner klin. Wochenschrift*, No. 2, January 14, 1895. Basing his reasoning upon the supposition that the respiratory failure under ether or chloroform narcosis is due to reflex disturbances excited by irritation of the nasal mucous membrane, he proposes the preliminary cocainization of this membrane. He claims that in practice he has noticed that anæsthesia is reached with less discomfort; that the stage of excitement is shortened if not altogether abolished; that vomiting is rarer; and that the unpleasant after-effects are entirely done away with. A few minutes before the narcosis is begun, a hand-ball spray apparatus is used to introduce about two centigrammes of a 10 per cent. solution of cocaine into each nostril. The spray is directed upward toward the superior meatus, and then directly backward. The application is repeated in three minutes, using half the quantity of the general anæsthetic then begun. The cocaine is applied every thirty minutes during the narcosis.—*Medicine.*

